# Review article laser-induced hyperthermia on graphene oxide composites

**DOI:** 10.1186/s12951-023-01956-6

**Published:** 2023-06-20

**Authors:** Laura González-Rodríguez, Sara Pérez-Davila, Miriam López-Álvarez, Stefano Chiussi, Julia Serra, Pío González

**Affiliations:** 1 Grupo de Novos Materiais, CINTECX, Universidade de Vigo, Vigo, 36310 Spain; 2 Galicia Sur Health Research Institute (IIS Galicia Sur), SERGAS-UVIGO, Vigo, 36213 Spain

**Keywords:** Hyperthermia, Photothermal therapy, Thermal dose, Near-infrared radiation, Graphene oxide, Reduced graphene oxide

## Abstract

**Background:**

Hyperthermia-based therapies have shown great potential for clinical applications such as for the antitumor and antipathogenic activities. Within all strategies, the so-called photothermal therapy proposes to induce the hyperthermia by the remote laser radiation on a photothermal conversion agent, in contact with the target tissue.

**Methods:**

This paper reviews the most relevant *in vitro* and *in vivo* studies focused on NIR laser-induced hyperthermia due to photoexcitation of graphene oxide (GO) and reduced graphene oxide (rGO). Relevant parameters such as the amount of GO/rGO, the influence of the laser wavelength and power density are considered. Moreover, the required temperature and exposure time for each antitumor/antipathogenic case are collected and unified in a thermal dose parameter: the CEM43.

**Results:**

The calculated CEM43 thermal doses revealed a great variability for the same type of tumor/strain. In order to detect potential tendencies, the values were classified into four ranges, varying from CEM43 < 60 min to CEM43 ≥ 1 year. Thus, a preference for moderate thermal doses of CEM43 < 1 year was detected in antitumor activity, with temperatures ≤ 50 °C and exposure time ≤ 15 min. In case of the antipathogenic studies, the most used thermal dose was higher, CEM43 ≥ 1 year, with ablative hyperthermia (> 60ºC).

**Conclusions:**

The ability of GO/rGO as effective photothermal conversion agents to promote a controlled hyperthermia is proven. The variability found for the CEM43 thermal doses on the reviewed studies reveals the potentiality to evaluate, for each application, the use of lower temperatures, by modulating time and/or repetitions in the doses.

**Supplementary Information:**

The online version contains supplementary material available at 10.1186/s12951-023-01956-6.

## Introduction

Hyperthermia refers to an increase in body temperature that can be induced by an external energy source, with the aim of achieving beneficial effects in the treatment of some pathologies and disorders [1]. Among the application strategies, local hyperthermia allows the treatment of small areas with sufficient preservation of healthy tissues [2]. Potential biological effects of hyperthermia were first proved by Eugene Robinson in 1974 demonstrating that heat had a selective cytotoxicity on hypoxic cells [3]. Several years later this selective cytotoxicity was discovered to occur specifically in some cell types, such as cancer cells [4], due to basic physiological differences between cancerous and healthy tissue vasculature. During the same period of time, high temperatures were also proven to inhibit bacterial growth, by affecting their motility and cell wall integrity [5]. In addition, Song *et al.* demonstrated in 1980 that there was a significant increase of blood flow in heated healthy tissues, indicating that hyperthermia can also induce an immune reaction or vascular stimulation, associated with the first proliferative phase of the healing process [6, 7].

According to the temperature ranges applied, induced hyperthermia can be classified [8–10] as: (i) mild, when the typical physiological temperature is exceeded only by a few degrees, being 43 °C the upper threshold considered for most authors; (ii) moderate, when the temperature ranges above this mild threshold but below 50 °C and (iii) the ablative hyperthermia, for temperatures in the 50–55 °C range, which produces the more severe effects.

Currently, most hyperthermia systems work by exposing the target tissue to energies generated by ultrasound (US) or electromagnetic (EM) radiation sources. Going into detail with electromagnetic radiation, the most commonly used systems are based on radiofrequency (RF), microwave (MW) and infrared (IR) [11]. Other strategies explore the possibility of using electromagnetic radiation to irradiate photosensitive materials with the resulting release of heat. This is the case of the so-called photothermal therapy (PTT), preferably mediated by radiation in the first and second near-infrared (NIR) windows, respectively located in the 650–950 nm (NIR-I) and 1000–1350 nm wavelength ranges (NIR-IIa). At these ranges the light presents its maximum depth of penetration into the biological tissues [12], being lasers emitting at 808 nm wavelength the most widely NIR radiation sources, less expensive in relation to the ones required for NIR-II. This strategy requires short time interactions with the target tissue, and provides with spatiotemporal addressability and minimal invasiveness in comparison to chemotherapy, photocatalytic and photodynamic therapy [13–16].

Regardless of the strategy selected to induce the hyperthermia, the application of the thermal treatment involves the contribution of two basic parameters: the required temperature to be reached at the biological tissue of interest, and the application time of interaction or exposure time. These two parameters, related by a normalized method, define the so-called thermal dose, which will determine the effectiveness of the induced hyperthermia as a treatment and will allow comparison between treatments. In relation to this, and given its great importance, several mathematical models have been proposed to express that time-temperature relationship. In particular, the use of CEM43 (cumulative equivalent minutes at 43 °C) stands out as a thermal dose method that finds its roots in the application of the Arrhenius model to study the kinetics of cell killing by hyperthermia. The CEM43 index, obtained using **Eq. 1**, converts the various time-temperature exposures applied into an equivalent exposure time at the reference temperature of 43ºC expressed as minutes, allowing to assess the amount of tissue damage caused by heat and, consequently, its use for clinical application [17, 18]. With this type of tools, despite the intrinsic mathematical limitations, it is able to refine the combination of exposure time and applied temperature, therefore to manage the thermal dosimetry, for reaching an effective but safe hyperthermia effect.

$$CEM43=\sum _{i=1}^{n}{t}_{i} . {R}^{(43-Ti)}$$

**Equation 1**. Formula for the calculation of the CEM43 parameter proposed by Dewhirst *et al.*, where *t*_*i*_ is the *i*-th time interval, *R* a constant related to the applied temperature (0.25 for *T* < 43 °C and 0.5 for *T* > 43 °C) and *T*_*i*_ the average temperature during time interval *t*_*i*_ [19].

With respect to the materials involved in photothermal therapy (PTT), graphene derivatives have been identified as photothermal conversion agents of interest, responsible for the light-to-heat conversion. More specifically, graphene oxide (GO) and reduced graphene oxide (rGO) are the most highly investigated in the biomedical field. Their oxygen content, number of layers and lateral dimensions provide GO and rGO with improved properties with respect to graphene such as better water suspending capacity, resulting in higher biocompatibility. Moreover, they present the ability to be tailored with additional functionalities, due to the presence of reactive groups such as carboxylic acid, epoxide, and hydroxide [20]. These characteristics make GO and rGO very versatile and simple to handle in aqueous media in addition to their low cost. In relation to their good ability to absorb NIR light [21–23], the photothermal conversion efficiency in the near-infrared wavelength region for graphene derivatives is estimated in about a 50%, very close to that of pure graphene and similar from those calculated for other photothermal agents [24, 25]. In the specific case of GO, Savchuk and collaborators [24] estimated an efficiency of 58 ± 5% when irradiated with a wavelength of 808 nm and a laser power of 200 mW. This value is higher than the one reported by the same authors for Au nanostructures and several semiconductor materials, polymer nanostructures or nanoparticles with ferromagnetic properties for the same wavelength.

The present paper reviews the most relevant studies based on the evaluation of induced hyperthermia triggered by NIR laser photoexcitation of GO and/or rGO as a potential effective strategy for antitumor and/or antipathogenic activities. Moreover, by using the **Eq. 1** above presented, the effective thermal dose CEM43, as a unifying parameter, has been determined for each of the reviewed studies, to be able to compare the obtained results for the two activities evaluated and, in this way, gain knowledge and visualize tendencies.

## Antitumor activity

As previously mentioned, hyperthermia has been proven to generate a selective cytotoxicity, by altering the integrity of certain cells at different levels (changes in membrane permeability, protein denaturation, specific HSPs release, DNA damage…). Within the cells investigated, this selectivity was also found for cancer cells, making the hyperthermia as a potential strategy to be followed in the treatment of localized tumors.

Table 1 summarizes the reviewed papers, and main parameters provided by the authors, on the evaluation of the antitumor efficacy of PTT mediated by NIR photoexcitation of GO/rGO [26–57]. The results are presented grouped by type of tumor cells evaluated, both in human and murine model lines, which are directly related to the most diagnosed cancers today: breast, cervix, prostate and lung cancer [58] and, therefore, extensively studied in all aspects. Thus, as it can be observed in Table 1, breast and cervical carcinoma represent almost half of the total number of papers reviewed [26–32, 38, 49, 52–57]. Prostate and lung cancer lines were also studied, although to a much lesser extent [33–35] and, finally, studies with osteosarcoma and other cancer cell lines are also included [36, 37, 39–47, 50, 51].

**Table 1:** Summary of the reviewed studies related to antitumor activity based on hyperthermia generated by photon excitation of GO and/or rGO in the NIR range

*In vitro* cell line		Material	Laser wavelength [nm]		Power density [W cm^− 2^]		Temperature reached [°C]	Exposure time [min]	CEM43 thermal dose [min]	Results (*in vitro* assays)	*In vivo* conditions	Results (*in vivo* assays)	Ref.
4T1 breast (murine model)	GO			808		2.0	48	3	36	No significant effect is achieved without a complementary drug	-		[27]
MCF-7 breast	GO			810		1.4	35	3	1.5 × 10^− 5^ (< 1 min)	No photothermal effect data on cells without chemotherapeutic agent	-		[55]
EMT6 breast (murine model)	GO			808		2.0	≈ 50	3	384 (6.4 h)	Cells inhibition rate of 80%	Tail vein injected 40 days treatment	PTT with GO presented the same effectiveness than the obtained with low-dose drugs, no regrow	[56]
MDA-MB-231 breast	GO			808		2.0	≈ 50	5	273 (4.5 h)	An 87% inhibition rate of cells	Tail vein injected 20 days treatment	Tumors completely inhibited, without recurrence	[26]
MDA-MB-231 breast	rGO			808		2.0	≈ 50	10	309 (5.1 h)	Viability of cancer cells significantly reduced (by about 25%)	-		[57]
MCF-7 breast	rGO			808		0.6	50	5	137 (2.3 h)	PTT effect dependent on the concentration of the material; total inhibition is achieved with ≥ 100 mg/L	Not specified	No apparent differences *in vivo* after a single dose of radiation	[28]
MCF-7 breast	GO and rGO			808		2.5	≈ 80	8	≈ 2 million years	GO exhibit excellent photothermal abilities capable to eliminate effectively the 95% of cancer cells	-		[29]
Hela cervical	rGO			808		0.2	39	10	0.002 (< 1 min)	Viability in cell line used decreases to about 60%	-		[30]
Hela cervical	GO			808		0.4	≈ 41	10	0.625 (< 1 min)	Very strong inhibition of tumor cells (significantly different from 25 µg/mL)	Tail vein injected 14 days treatment	Effective tumor PTT with only one irradiation	[31]
Hela cervical	rGO			808		-	65	7	4.3 × 10^6^ (8.2 years)	Significant elimination of cancer cells	-		[32]
Hela cervical	rGO			808		4.0	52	5	2064 (1.4 days)	Significant differences in cancer cell death (reduction of about 15%)	-		[38]
Hela cervical	GO			808		5.0	44	3	2	Significant decrease in cell viability	-		[49]
Hela cervical	rGO			808		0.6	≈ 55	10	1.1 × 10^4^ (7.6 days)	Significant differences in cancer cell death at the highest concentration. Death of cells > 50%	-		[52]
Hela cervical	GO			808		0.72	63	10	1.1 × 10^7^ (19.9 years)	Up to 60% reduction in cell viability	Intravenously injected	Noticeable reduction in the tumor size	[53]
Hela cervical	GO			808		2.0	79	10	6.8 × 10^11^ (≈ 1 million years)	More than 50% reduction in cell viability	Intratumorally injected 14 days treatment	The tumor disappeared completely	[54]
A549 lung	rGO			808		2.0	≈ 65	5	8.9 × 10^6^ (16.9 years)	When the maximum non-cytotoxic concentration is irradiated, cancer cells viability is reduced by up to 40% and normal cells are maintained at 70%	-		[35]
PC3 prostate	GO			808		3.0	45	5	4	Trend of cells elimination but no significant differences	Intratumorally injected 15 days treatment	No improvement but tumor volume is maintained and does not grow	[33]
LNCaP prostate	GO and rGO			808		7.5	45	1	4	Decrease in cancer cells viability much more pronounced when using rGO. Almost a total death is achieved	-		[34]
HOS osteosarcoma	GO			808		2	48	5	160 (2.6 h)	The scaffold showed excellent photothermal response *in vitro*; 30% of cells in apoptosis	Scaffold implantation	The tumor volume changed and and an extensive necrosis effect appeared	[36]
MG-63 osteosarcoma	GO			808		1.5	45	8	33	Differences in viability of cells at longer exposure times	-		[37]
OE-19 esophageal	GO and rGO			808		3.0	≈ 40	10	0.05 (< 1 min)	No significant effects	-		[51]
C26 colon	rGO			785		9.62	≈ 42	10	0.77 (< 1 min)	No significant effect on cell proliferation is achieved without a complementary photothermal agent	-		[39]
KM12C colon	GO			808		0.3	≈ 42	20	1	Almost complete inhibition of cells	-		[41]
CT26 colon (murine model)	rGO			808		1.0	53	8	2340 (1.6 days)	Over 80% of cancer cells reduction	Subcutaneously injected 16 days treatment	Activation of the immune response	[44]
BEL-7402 hepatoma K-150 esophageal HCT-116 colon	GO			808		2.0	≈ 48	5	36	Inhibition of cell growth in all lines but strongest for hepatoma	Tail vein injected	Significant differences in tumor volume but without being eradicated	[47]
B16F10 melanoma (murine model)	GO and rGO			960		2	≈ 43	60	60	Cell death was confirmed by the release of lactate dehydrogenase from dead and dying tumor cells	-		[42]
A-431 epidermoid carcinoma	rGO			810		0.15	≈ 45	30	741 (12.3 h)	Cancer cells viability is reduced by up to 60%	-		[43]
B16F10 melanoma (murine model)	GO			808		2.0	≈ 50	10	1280 (21.3 h)	Drastic decrease of cells viability from 200 µg/mL and above	Topically administered	Complete ablation of tumor tissues with no recurrence	[45]
U87 MG glioblastoma	GO			808		2.0	43	3	1	Significant results in the elimination of cancer cells	-		[40]
U87 MG glioblastoma	GO and rGO			808		0.6	55	8	14,884 (10.3 days)	Better photothermal capacity of rGO compared to GO. Drastic reduction of viability	-		[46]
U87 MG glioblastoma	rGO			808		2.5	≈ 55	10	9024 (6.3 days)	80% cell death is achieved	-		[50]

In terms of the material and laser parameters, the concentration of GO/rGO evaluated in all the mentioned studies was of up to 1 mg/mL and with a preferential use of laser emitting in 808 nm. The exceptions for wavelength correspond generally to the use of others close to 808 nm, within the NIR first biological window [39, 42, 43, 55]. However, in recent years, wavelengths in the second NIR window have been explored, especially in the NIR-IIa window (1300–1400 nm) given its superiority in penetration depth and maximum permissible exposure over NIR-I window. Thus, Xu *et al.* [59] evaluated the use of laser excitation at 1275 nm demonstrating that it was practicable and exhibited, according to the authors, much more desirable outcomes in deep-tissue antitumor capabilities* in vivo* compared to that of 808 nm laser. Polyethylene glycol-stabilized copper sulfide nanoparticles were the photothermal conversion agents used at this study. Reference should also be made to the laser powers used, the influence of which was proposed by Vila *et al.* [60]. Thus, these authors concluded that after irradiating osteosarcoma cell line with internalized GO, the cell culture temperature increases preferentially with laser power (from 1.5 to 3 W cm^− 2^) rather than with exposure time (from 1 to 7 min). Moreover, for the highest tested laser powers, necrosis was the preferential cell death, leading to an increment in cytokine release to the medium. In relation to this, it stands out that most of the reviewed studies use a wide range of power densities to determine the most adequate value for subsequent tests. In this way, most of them applied a power density lower than 5 W cm^− 2^, and only two studies, among the oldest ones, used higher power densities of up to approximately 9 W cm^− 2^ [34, 39].

When considering the parameters of temperature reached and exposure time (Table 1), the evaluation of antitumor efficacy revealed a great variability, with temperature ranging from mild hyperthermia, close to 40ºC, to ablative by applying even very high temperatures, up to 80ºC, widely exceeding therefore the 55ºC. In percentage, a 25% of the reviewed works used a temperature ≥ 55ºC. In the case of the exposure time, something similar happens, varying from minutes to one hour. At this point, due to the aforementioned variability on these both parameters, and in order to be able to compare the effectiveness of the results, the reviewed data have been unified by calculating the corresponding CEM43 thermal dose. This value has been also incorporated to Table 1 as increasing thermal doses applied for the same type of cancer cells. On this basis, a great variability in CEM43 thermal doses is clearly observed for each type of cancer cells, with values from less than a minute to millions of years. In line with this, four ranges of CEM43 thermal dose are then proposed:

- CEM43 < 60 min, thermal dose in the range of minutes. These studies did not find significant differences in terms of cell viability [27, 30, 31, 33, 36, 39–41, 51, 55], with the notable exception of the ones that used the Hela cell line (cervical carcinoma) [30–32].
- 1 h ≤ CEM43 < 24 h, thermal dose in the range of hours. In this case the results of these studies demonstrated a reduction of tumor cells for all the lines tested [26, 37, 38, 42–45, 56, 57]. However, variability was detected in the results in terms of viability, e.g. four of these studies quantified a tumor cells viability reduction higher than 60% [26, 43, 44, 56], while Kang *et al.* and Mun *et al.* obtained a lower reduction with values in the range of 15–25% [38, 57].
- 1 day ≤ CEM43 < 365 days, thermal dose in the range of days. The results of these studies showed an effective inhibition of cancer cells viability using this higher thermal dose, with a reduction of cell viability of more than 50% in all cases [28, 34, 46, 49, 52], even reaching almost total inhibition or direct necrosis in some studies [28, 34, 46].
- CEM43 ≥ 1 year, thermal dose in the range of years. In these studies, the applied temperature exceeded 60ºC, which supposes a very high thermal dose with powerful results in terms of cell death [29, 32, 35, 50, 53, 54].

With these four ranges in mind, it can be seen that the majority of the reviewed *in vitro* studies have selected medium thermal doses, situated in the first two ranges established: CEM43 < 60 min and 1 h ≤ CEM43 < 24 h. Despite this clear trend towards the use of CEM43 thermal doses of less than a day or few days, there is variability in the results obtained influenced, not only by the hyperthermia conditions applied, but also by the inherent differences between the cell lines. The relevance of the stage of the cell cycle and the culture conditions must be also taken into account together with the thermo-tolerance that mammalian cells develop after short periods of exposure to moderate temperatures or longer periods of exposure to sub-lethal temperatures [61–63]. However, no specific differences can be elucidated depending on the origin of the cell line used.

With respect to the *in vivo* tests, a remarkable number of the reviewed studies completed their research with ectopic xenograft tumor models in small rodent, using the same cell type than *in vitro* assays. These ones have been also identified in Table 1. In these tests, apart from the PTT conditions applied and the strategy followed, the route of administration of the photothermal conversion agents seems to be of special relevance, being the intravenous and the intratumoral the two most common routes to administer the GO/rGO. There were exceptions such as in the research work carried out by Yan *et al.* where the subcutaneous route [44] was chosen; or at the *in vivo* evaluation presented by Sang Jung *et al. *where the compound was applied topically because a certain type of skin cancer was investigated [45]. Moreover, following a different strategy, Liang Ma *et al.* manufactured a scaffold that was incorporated directly into the tumor [36]. The overall analysis of the reviewed studies where an *in vivo* evaluation was carried out, shown at Table 1, revealed that the antitumor effectiveness achieved was mainly established based on the assessment of two parameters: variation in the tumor volume and in the animals’ total weight. Within them, two studies [26, 45] indicated a total inhibition of the tumor, complete reduction in tumor volume, concluding that there was no recurrence during the following weeks. In these two cases, GO was the photothermal conversion agent used with laser conditions of 808 nm wavelength and 2 W cm^− 2^ power administered, by different routes, resulting in an applied thermal dose CEM43 of 273 min in case of breast cancer and of 1280 min in melanoma. In both cases de CEM43 remained below 24 h, in the 1 h < CEM43 < 24 h, thermal dose range. There was another study, Zhang *et al.* [56], that proposed a combination of chemotherapy and photothermal therapy in one system, using graphene oxide as photothermal agent but also as a delivery agent of a conventional antitumor drug for breast tumor. The results demonstrated that the synergistic effect significantly improved the therapeutic efficacy, and presented a lower toxicity when compared to the administration of the antitumor agent alone. The calculated CEM43 thermal dose applied at this case, where photothermal therapy was combined with chemotherapy, was lower with a value of 384 min, again in the range 1 h < CEM43 < 24 h range.

## Antipathogenic activity

Infections caused by some bacterial and fungal strains are attracting great concern in the medical community because of the high mortality rates associated. Within them, Kraker *et al.* [64] highlighted the mortality and consequences caused by two particular bacterial strains: methicillin-resistant *Staphylococcus aureus* (MRSA) and third-generation cephalosporin-resistant *Escherichia coli* (G3CREC). This high mortality is due to the decreasing effectiveness of conventional treatments against these microorganisms, a process that has increased in recent decades due to various factors such as the misuse of antibiotics. This resistance, identified as one of the top 10 global public health threats [65], urges the development of alternative procedures to fight the so-called multi-drug resistant pathogens, being hyperthermia-based systems an interesting approach.

Going into detail, the two mechanisms of action that alter the integrity of microorganisms are the membrane disruption and oxidative stress. Both processes can be caused by localized hyperthermia generated by NIR irradiation of GO/rGO [66]. The first effect (membrane disruption) is the result of physical damage produced by the GO/rGO nanosheets themselves. This leads to instability of the cellular structure and consequently the bacteria inactivation [67]. The second effect, oxidative stress, occurs at the same time, due to the reactive oxygen species (ROS) produced by these graphene derivatives. This causes damage to cellular components, such as lipids and proteins, and with them the mitochondrial dysfunction and DNA damage, after being internalized by cells [68]. Both effects are increased by the additional damages caused by hyperthermia in the same way [69].

Table 2 collects a summary of the reviewed publications, with main parameters provided by the authors, on the evaluation of the antimicrobial efficacy of PTT mediated by NIR photoexcitation of GO/rGO [70–78]. Additionally, the CEM43 thermal dose has also been calculated, and incorporated for each publication. Thus, when first looking at Table 2, it is noted that the studies are mainly focused on the most investigated bacterial strains, above mentioned, *Staphylococcus aureus* and *Escherichia coli*. However, two of the reviewed publications [70, 71] include studies with pathogenic fungi such as *Saccharomyces cerevisiae* and some species of the genus *Candida*. This latter is also considered already at the same threat level as the assigned to the above-mentioned bacteria given several evidences of drug-resistant *Candida* yeasts [79]. In relation to the photothermal conversion agents, GO or rGO have been used mostly as nanosheets and nanotubes in dispersions or scaffolds. Moreover, it is also important to take into account the antimicrobial action derived solely from the presence of these graphene derivatives, already proven at previous studies [80, 81] where authors concluded that GO produces the strongest effect, followed by rGO, graphite and graphite oxide. When hyperthermia is additionally incorporated, induced by PTT using the GO/rGO as PTCAs, the reviewed publications presented at Table 2 revealed that none of the studies used GO/rGO concentrations exceeding 100 µg/mL. When considering the laser parameters, almost all authors used a wavelength of 808 nm, as well as in the antitumor activity, which is within the *first biological window*. Two of the reviewed works [71, 76] applied different wavelengths, 660 and 1064 nm respectively, which although not the most studied wavelengths, they both still belong to the NIR biological window. Furthermore, in relation to the power laser, it seems that the trend is moving towards the use of soft power densities ≤ 1.5 W cm^− 2^ and, to our best knowledge, only two reviewed studies exceeded this value, using up to 3 W cm^− 2^ [72, 73]. When considering the temperature, a clear tendency was detected on these antipathogenic studies with the application of high temperatures in the ablative range with values ≥ 55ºC in the 100% of the cases.

**Table 2:** Summary of the reviewed studies related to antipathogenic activity based on hyperthermia generated by photon excitation of GO and/or rGO in the NIR range

Type of microorganism	Material	Laser Wavelength [nm]	Power Density [W cm^− 2^]	Temperature reached [°C]	Exposure Time [min]	CEM43 thermal dose [min]	*In vivo*	Results	Ref.
*S. aureus*	rGO	808	0.4	60	10	1.3 × 10^6^ (2.5 years)	-	Almost 100% killing efficiency and maintained even after storage for 30 days	[77]
*E. coli*	GO	808	2.78	65	5	2.1 × 10^7^ (39.9 years)	-	Total antibacterial capability > 98%	[73]
*S. aureus* and *E. coli*	GO	808	1.5	55	5	5281 (3.6 days)	-	Almost complete elimination of bacteria	[74]
*S. aureus* and *E. coli*	GO	808	0.6	55	60	2.4 × 10^5^ (170 days)	-	Antibacterial effect of 99.91%	[75]
*S. aureus* and *E. coli*	GO	660	0.9	65	10	2.7 × 10^7^ (52.3 years)	14 days treatment	Significant differences in antibacterial potential of up to 54% for *S. aureus* and 66% for *E. coli*	[76]
*S. aureus* and *E. coli*	rGO	808	3.0	70	6	8.0 × 10^8^ (thousands of years)	-	Bacterial viability reduced to 0%	[72]
*E. coli* and *S. typhimurium*	GO	808	-	66	15	6.4 × 10^7^ (122.2 years)	-	Effective killing (*>* 95%)	[78]
*S. aureus* and *P. aeruginosa* *S. cerevisiae* and *C. albicans*	GO	808	1.0	61	6	1.6 × 10^6^ (2.9 years)	21 days treatment	Significant differences *in vitro* and *in vivo* results. Topical GO incorporation and daily irradiation had effective potential	[70]
*S. aureus* and *P. aeruginosa* *S. cerevisiae* and *C. utilis*	GO	1064	-	64	3	6.3 × 10^6^ (11.9 years)	6 days treatment	Not only the *in vivo* infection is stopped but also the wound healing is improved	[71]

As in the studies for tumor cells, the thermal dose has also been calculated for the antipathogenic reviewed works presented at Table 2, using the CEM43 parameter. It can be observed (Table 2) that the thermal doses applied are very high, 77.8% in the range CEM43 ≥ 1 year [70–73, 76–78] and 22.2% in the 1 day ≤ CEM43 < 365 days’ range [74, 75]. These high thermal doses are generated by the use of high temperatures, included in the ablation range (≥ 55ºC), combined with exposure times of great variability from 3 to even 60 min. The use of such high temperatures can be justified by the recently demonstrated effect on the morphology of the biofilm formed by *Staphylococcus* bacteria when exposed to local heating above 60 °C and for long periods of time [82]. According to the biological response to these thermal doses, a very strong decrease in microbial viability was achieved in all *in vitro* tests. However, only three of the reviewed works carried out animal experiments and offered promising results with an enhanced reduction of bacterial infection [70, 71, 76]. Moreover, these three *in vivo* studies observed a simultaneous acceleration of the wound healing, with faster healing of skin wounds compared to the control probes in both, mouse and rat populations. Additionally, Li and collaborators [76] claimed the absence of toxicity of their strategy, based on a composite with Zn and GO and dual light irradiation for 10 min, to organs and proposed some regulatory mechanisms that may occur. Finally, clear positive antipathogenic results were observed from the first days of treatment by Wu *et al.* [70], who also claimed that the injuries of rats treated with the GO composite did not show erythema symptoms.

## Concluding remarks and future perspectives

**Fig. 1:**
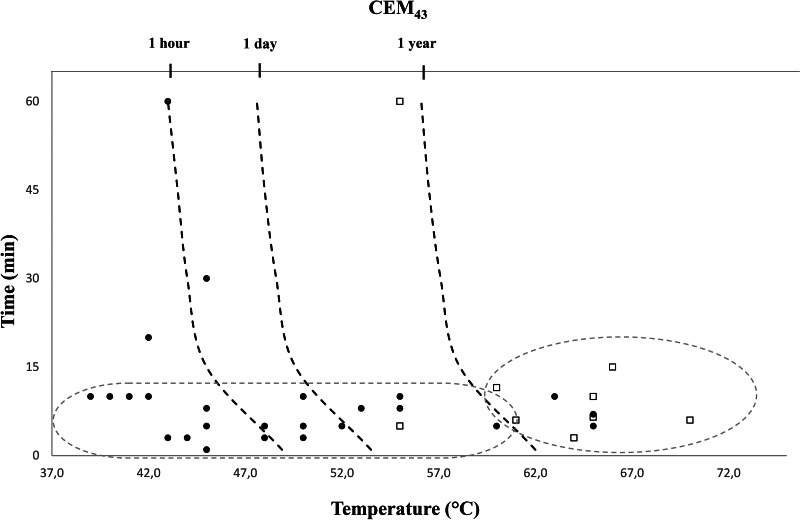
Graph showing the different ranges of CEM43 thermal dose and the position of those used by all reviewed studies, calculated according to Eq. 1. The dotted ovals encompass the thermal doses used by most of the reviewed studies, with antitumor application represented by black spots and antipathogenic studies represented by white squares

As clearly documented in the present review, both graphene derivatives, GO and rGO, show a remarkable ability as effective photothermal agents when irradiated with NIR lasers and can be used in a controlled hyperthermia process. Moreover, based on the data of temperature and exposure time collected from the reviewed studies, the unified thermal dose parameter CEM43 allowed differentiating four ranges of interaction heat-cell in three thresholds: 1 h, 1 day and 1 year. Fig 1 presents the CEM43 thermal dose ranges established based on these three thresholds: CEM43 < 60 min, 1 h ≤ CEM43 < 24 h, 1 day ≤ CEM43 < 365 days and CEM43 ≥ 1 year, together with the position of the thermal dose calculated for each of the reviewed studies. From it, despite the variability, the preference for a certain thermal dose depending on the cell type to be irradiated (tumor cell/pathogen) is revealed. Being the moderate thermal doses of CEM43 < 1 year the most used for antitumor activity (dotted oval: black spots), with most temperatures ≤ 50 °C and exposure time ≤ 15 min. While, in case of the antipathogenic studies (dotted oval: white squares), the most used thermal dose was higher, CEM43 ≥ 1 year, with ablative hyperthermia (> 60ºC). Finally, observing those two tendencies and each range values, the interest of extended research on the application of lower thermal doses: CEM43 < 1 h for antitumor activity and CEM43 < 1 year for antipathogenic activity is clear. The efficient application of lower temperatures by modulating the thermal dose with the time of exposure and/or repetitions in the doses will also contribute to reduce the damage on surrounding tissues.

## Electronic supplementary material

Below is the link to the electronic supplementary material.

Supplementary Material 1

## Data Availability

Not applicable.
