# Supplementary figures and images for: Review article laser-induced hyperthermia on graphene oxide composites
Source: J Nanobiotechnology. 2023 Jun 20;21:196. doi: 10.1186/s12951-023-01956-6 (PMC10280920; doi:10.1186/s12951-023-01956-6)

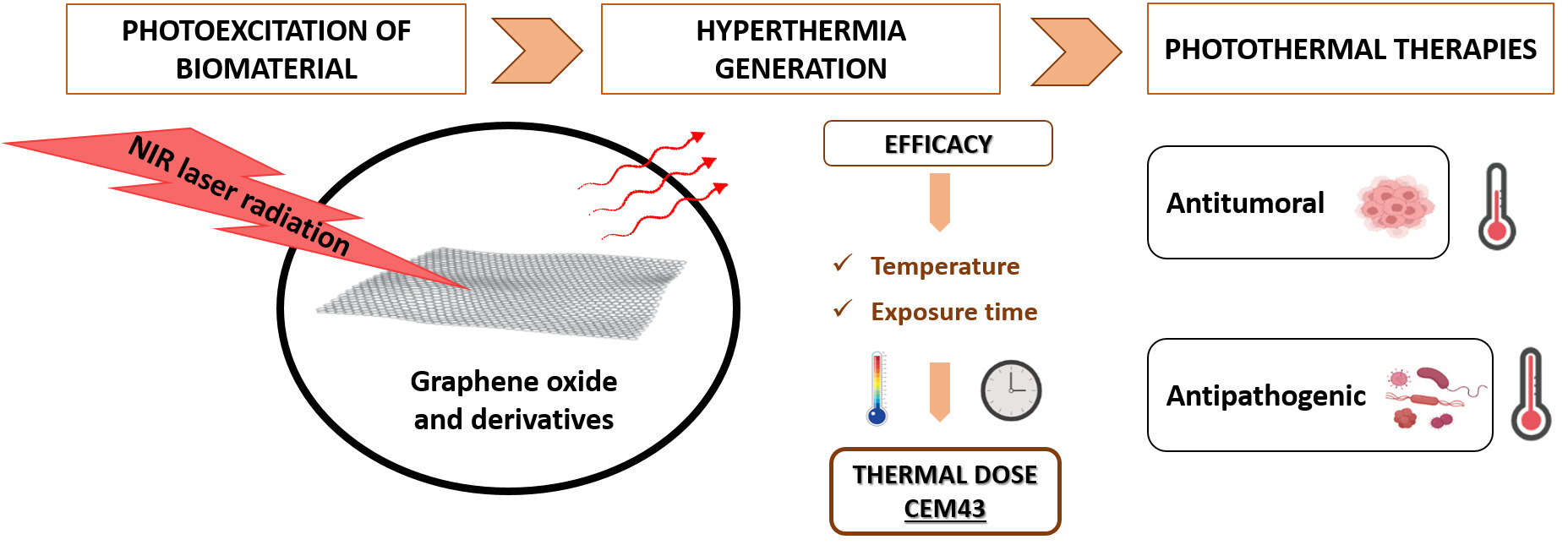

Supplement: Supplementary file 1 — Supplementary Material 1 [file 12951_2023_1956_MOESM1_ESM.jpg]
